# Visualization of the entire process of rice spikelet infection by *Ustilaginoidea virens* through nondestructive inoculation

**DOI:** 10.3389/fmicb.2023.1228597

**Published:** 2023-08-10

**Authors:** Xianfeng Hu, Jian Wang, Yubo Zhang, Xiaomao Wu, Rongyu Li, Ming Li

**Affiliations:** ^1^College of Agriculture, Anshun University, Anshun, Guizhou, China; ^2^Institute of Crop Protection, Guizhou University, Guiyang, Guizhou, China; ^3^College of Agriculture, Guizhou University, Guiyang, Guizhou, China; ^4^Provincial Key Laboratory for Agricultural Pest Management in Mountainous Region, Guizhou University, Guiyang, Guizhou, China

**Keywords:** *Ustilaginoidea virens*, rice false smut, infection process, visualization, nondestructive inoculation

## Abstract

**Introduction:**

Rice false smut caused by *Ustilaginoidea virens*, is a destructive fungal disease encountered in many rice-producing areas worldwide. To determine the process by which *U. virens* infects rice spikelets in the field.

**Methods:**

The green fluorescent protein-labeled *U. virens* was used as an inoculum to conduct artificial inoculation on rice at the booting stage via non-destructive panicle sheath instillation inoculation.

**Results:**

The results showed that the conidia of *U. virens* germinated on the surface of rice glumes and produced hyphae, which clustered at the mouth of rice glumes and entered the glumes through the gap between the palea and lemma. The conidia of *U. virens* colonized in rice floral organs, which led to pollen abortion of rice. *U. virens* wrapped the whole rice floral organ, and the floral organ-hyphae complex gradually expanded to open the glumes to form a rice false smut ball, which was two to three times larger than that observed in normal rice.

**Discussion:**

Panicle sheath instillation inoculation was shown to be a non-destructive inoculation method that could simulate the natural infection of *U. virens* in the field. The entire infection process of *U. virens* was visualized, providing a theoretical reference for formulating strategies to control rice false smut in the field.

## 1. Introduction

Rice false smut is a fungal disease prevalent in rice-producing regions worldwide, and is caused by *Ustilaginoidea virens* that infects the rice panicles (Tanaka et al., 2008; Zhang et al., 2014; Wei et al., 2020). In recent years, due to the large-scale promotion of high-yield hybrid varieties, excessive application of chemical fertilizers, and impact of climate change, rice false smut has transitioned from a minor disease to a major disease affecting current rice production (Tang et al., 2013). Rice false smut not only seriously decreases the yield and quality of rice, but also produces ustiloxin toxins which is toxic to human beings and animals, thus affecting the rice safety (Koiso et al., 1992; Miyazaki et al., 2009; Shan et al., 2012; Fu et al., 2015; Li et al., 2019). This fungus also produces ustilaginoidins, a class of bis-naphthol-γ-pyrrolone compounds with inhibitory effects on radicle elongation cells of rice seeds (Sun et al., 2016). Since the mycelial growth of *U. virens* is slow under artificial culture conditions and the occurrence of rice false smut is greatly affected by the environmental conditions, artificial inoculation of *U. virens* is not satisfactory (Guo et al., 2012; He et al., 2015). At present, injection inoculation is the widely used to determine the pathogenicity of *U. virens* and evaluate the resistance level of rice to rice false smut (Zhou et al., 2014; Han et al., 2015). In this method, a mixture of mycelia and conidia of *U. virens* is injected into the panicle sheath of rice at the booting stage to induce rice false smut (Chao et al., 2017). The rate of diseased panicles of rice at the late booting stage was found to be higher than that other times. The inoculation temperature and humidity have great influence on the success rate of diseased panicles. The rate of diseased panicles upon inoculation at 20°C is significantly lower than that upon inoculation at 28°C (Jia et al., 2015). In addition, appropriate low temperature stimulation and moisture treatment can promote infection by *U. virens* (Ashizawa et al., 2011). Nevertheless, injection inoculation damages the panicle sheath of rice that acts as a physical barrier. Therefore, it is difficult to explain how *U. virens* enters the panicle bud of rice in the field conditions by injection inoculation (Sun et al., 2020). Establishing an efficient and stable inoculation method to simulate the natural infection process of *U. virens* would be conducive to studying the behavior of *U. virens* in the field (Fan et al., 2016; Tanaka et al., 2017).

To achieve targeted prevention and control, it is essential to determine the natural infection process of *U. virens* in the field. In our previous studies, panicle sheath instillation did not destroy the structure of rice and successfully produced rice false smut balls, indicating that it is a nondestructive inoculation technique (Hu et al., 2021). Comparison between panicle sheath instillation and injection showed that the rice false balls formed via panicle sheath instillation and natural infection were mainly distributed in the middle and lower parts of the rice panicle, whereas those formed via injection were mainly distributed in the middle and upper parts of the rice panicle (Hu et al., 2021). Thus, panicle sheath instillation was considered as the inoculation technique to simulate the natural infection of *U. virens*, and it is speculated that *U. virens* in the field can enter the panicle sheath through the gap in the panicle sheath to complete infection (Hu et al., 2021). However, the infection process of *U. virens* inoculated using the panicle sheath drip method remains unclear. To visualize the entire infection process of *U. virens*, the rice panicle was inoculated with *U. virens* labeled with green fluorescent protein at the booting stage using panicle sheath instillation. This study will provide a theoretical reference for formulating strategies to control rice false smut.

## 2. Materials and methods

### 2.1. Strains of *U. virens*

The wild-type *U. virens* UV-PD11 strain and the green fluorescent protein-labeled *U. virens* gfp-z1, gfp-z2, and gfp-z3 strains were stored at the Institute of Crop Protection, Guizhou University.

### 2.2. Preparation of culture medium

Potato sucrose agar medium (PSA) and potato sucrose medium (PSB) were prepared. A total of 200 g of fresh potatoes were cut into thin slices, boiled for 30 min, filtered with three layers of gauze, and supplemented with 20 g of sucrose and 20 g of agar powder in a beaker with 1,000 ml of purified water. The medium were sterilized using a high-pressure steam sterilization pot (Shanghai ShenAn Medical Equipment Factory, Shanghai, China) at 121°C for 30 min. The procedure for preparing PSB medium was the same as that for preparing PSA medium, except that agar powder was not added.

### 2.3. Colony morphology and growth rate

The UV-PD11, gfp-z1, gfp-z2, and gfp-z3 strains were each inoculated into PSA medium. After 10 days, the colony diameter was recorded and the average growth rate was calculated using the following Equation 1.


(1) Average⁢growth⁢rate=Average⁢colony⁢diameter/Time

### 2.4. Rice cultivation

The seeds of the indica rice variety ‘Jinyou 785’ (Guizhou Jinnong Technology Co., Ltd., Guizhou, China) were disinfected with 75% ethanol (Shandong Chituma Medical Treatment Technology Co., Ltd., Shandong, China) and rinsed thoroughly with sterile water. Field management was carried out according to conventional rice cultivation measures, and fungicides were not used in the experimental area.

### 2.5. Nondestructive artificial inoculation method

The panicle sheath instillation was performed as follows: 5 cm PSA medium of *U. virens* was inoculated into 100 ml of PSB medium and cultured under shaking at 28°C and 130 rpm for 6 days. The mycelium of *U. virens* was ground and filtered with three layers of gauze to obtain hypha spore suspension. The filtrate was diluted with PSB medium to a final concentration of 1 × 10^6^ spores/ml hypha spore suspension as the inoculum. Inoculation was performed at stages 6–8 of young panicle differentiation of rice, with each inoculum having a volume of 3 ml. The filtrate was dripped into the panicle sheath of rice at a rate of 40 drops per minute. The rice panicle sheath was wrapped with a plastic film for heat and moisture preservation, which was removed after 3 days.

### 2.6. Visualization of the infection process of *U. virens*

The rice variety ‘Jinyou 785’ was inoculated with the hypha spore suspension of the strains gfp-z1, gfp-z2, gfp-z3, and UV-PD11 using panicle sheath instillation. The samples were taken at 3, 6, 8, 12, 15, and 18 days, and the spikelets on the rice panicles were cut. A single spikelet was cut with a double-edged blade and observed under a fluorescence microscope. The fluorescence light source of the microscope was turned on, and the excitation block was turned to the blue light source (wavelength 450–480 nm) to observe the colonization and expansion of *U. virens* in the rice floral organs (filament, stigma, style, and ovary). Each treatment included 7 rice plants and 3 replicates, with a total of 21 plants. The average rate of diseased panicles was calculated using Equation 2, and the significance was analyzed.


(2) $${\text{P}}_{\text{d}}={\text{N}}_{\text{d}}/{\text{N}}_{\text{t}}\times 100\%$$

P_*d*_, diseased panicle rate; N_*d*_, number of diseased panicles; N_*t*_, total number of panicles.

### 2.7. Reisolation and fluorescence detection of *U. virens*

The bright green fluorescence of the rice false smut balls could be detected under a fluorescence microscope (Ningbo Yongxin Optical Co., Ltd., Ningbo, China), which was selected as the isolation material. The rice false smut balls were clamped with sterile tweezers, and the chlamydospore powder was knocked down to the surface of the rifampicin-containing PSA medium by tapping the tweezers. The culture medium was cultured in the dark at a constant temperature (28°C) for 3–4 days, and then white-spot colonies were observed on the surface of the PSA culture medium. These colonies were picked out and placed in fresh PSA medium after 12 days. The morphology of the colonies was observed, and the fluorescence intensity was detected under a fluorescence microscope. The labeled hyphae of *U. virens* were picked out with a sterile inoculation needle and gently applied to the center of the groove of the glass slide. After the cover glass was covered, water around the cover glass was removed with absorbent paper and observed under a fluorescence microscope. A high-pressure mercury lamp was turned on and preheated for 15 min before performing the observations. *U. virens* was observed under a low-power bright field and with the bright-field light source turned off. The fluorescent light source of the fluorescence microscope was then turned on and the blue light source (wavelength 450–480 nm) was selected using the excitation block dial.

### 2.8. Statistical analysis

DPS software was used to analyze the variance of the experimental results. Here, *p* < 0.05 was considered to reflect a significant difference, whereas *p* < 0.01 was considered to reflect a very significant difference.

## 3. Results

### 3.1. Colony morphology of *U. virens*

The morphological characteristics and growth rates of the wild-type strain UV-PD11 and green fluorescent protein-labeled strains gfp-z1, gfp-z2, and gfp-z3 were measured to determine whether there were differences in the biological characteristics. The colony of UV-PD11 was generally round in shape with dense white hyphae, a raised center resembling a hat, and bright yellow color at the back, which turned dark green at the late stage of culture. The colonies of the strains gfp-z1, gfp-z2, and gfp-z3 were round overall and the hyphae were dense. The colony back of the green fluorescent protein-labeled strains was bright yellow, which were similar to those of the *U. virens* strain UV-PD11. There were no significant difference in the growth rate among the strains gfp-z1, gfp-z2, gfp-z3, and UV-PD11, indicating that there was no significant differences in morphology and growth rate between strains labeled with green fluorescent protein and wild-type strains (Supplementary Figure 1).

### 3.2. Mycelial morphology of *U. virens*

The growth of the apical hyphae of the strains gfp-z1, gfp-z2, gfp-z3, and UV-PD11 was compared to determine the morphological differences between the obtained transformants and the wild-type strain UV-PD11. The results showed that the hyphae of the strains gfp-z1, gfp-z2, gfp-z3, and UV-PD11 were pale yellow. There was no significant difference in hyphal thickness and diaphragm number of *U. virens*, and many branches were bifurcated (Supplementary Figure 2). Therefore, there was no significant difference in hyphal morphology between the strains gfp-z1, gfp-z2, and gfp-z3 and the wild-type strain UV-PD11.

### 3.3. Pathogenicity difference in *U. virens*

To explore the changes in the pathogenicity of *U. virens*, the rice variety ‘Jinyou 785’ was inoculated with the strains gfp-z1, gfp-z2, gfp-z3, and UV-PD11 using panicle sheath instillation. The average rate of diseased panicles was determined, and the significance of its differences among the strains was analyzed. The average rates of infected panicles of the strains gfp-z1, gfp-z2, gfp-z3, and UV-PD11 were 90.48, 80.95, 85.71, and 90.48%, respectively. There was no significant difference in pathogenicity among the strains gfp-z1, gfp-z2, gfp-z3, and UV-PD11 (Supplementary Figure 3).

### 3.4. Visualization of the infection process of *U. virens*

#### 3.4.1. Colonization of *U. virens* on the rice glumes

**FIGURE 1:**
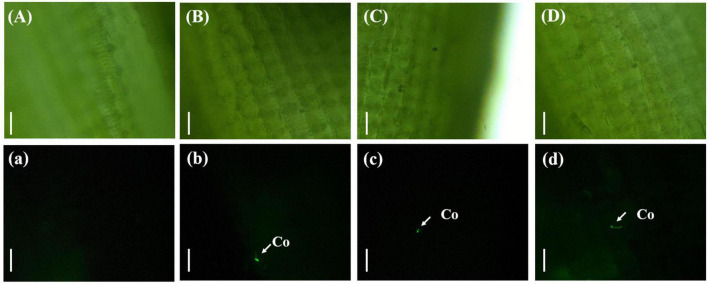
Single spore germination of *Ustilaginoidea virens* on the surface of the rice glume. **(Aa)** UV-PD11 strains, **(Bb,Cc,Dd)** gfp-z1 strain. **(A–D)** Bright field, **(a–d)** dark field. Co, conidia. Bar, 50 μm.

The rice was inoculated with gfp-z1 strain after 12 h, which were sampled and observed. It was difficult to observe the conidia of *U. virens* on the surface of the glumes under bright-field conditions (Figure 1). However, the conidia of *U. virens* could be observed in the dark field observation, and some conidia began to germinate and form hyphae under the excitation of blue light from the fluorescence microscope.

#### 3.4.2. Clustering of *U. virens* between the palea and lemma

**FIGURE 2:**
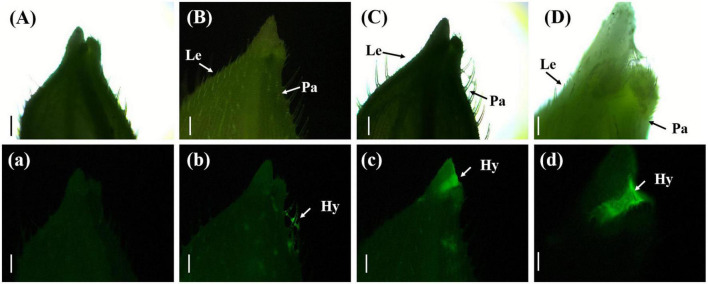
Aggregation of *Ustilaginoidea virens* at the gap between the palea and lemma. **(Aa)** UV-PD11 strains, **(Bb,Cc,Dd)** gfp-z1 strain. **(A–D)** Bright field, **(a–d)** dark field. Hy, hypha; Le, lemma; Pa, palea. Bars, 500 μm **(Aa,Bb,Cc)**, 250 μm **(Dd)**.

Three days after inoculation with gfp-z1 strain, the glume samples of rice were observed under an upright fluorescence microscope (Ningbo Yongxin Optical Co., Ltd., Ningbo, China) (Figure 2). It was found that a large number of conidia of *U. virens* on the surface of glume began to germinate, spread, and extend. Meanwhile, the gap between palea and lemma in rice and the awn around the spikelet shell emitted bright fluorescence, which indicated that numerous hyphae of *U. virens* had clustered there. This might have been due to the special structure of the gap between the palea and lemma, which was conducive to the attachment and colonization of *U. virens*. *U. virens* might enter the glume through the space between the palea and lemma of rice.

#### 3.4.3. Entry of *U. virens* into the rice glume

**FIGURE 3:**
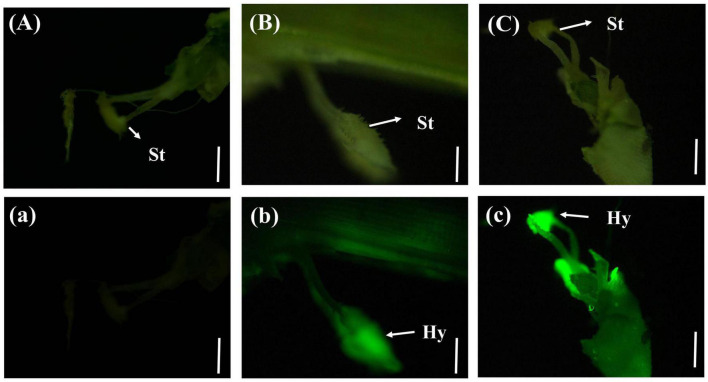
Colonization of *Ustilaginoidea virens* in floral organs. **(Aa)** UV-PD11 strains, **(Bb,Cc)** gfp-z1 strain. **(A–C)** Bright field, **(a–c)** dark field. Hy, hypha; St, stigma. Bars, 200 μm **(Aa,Bb)**, 500 μm **(Cc)**.

In a few rice stigmas, fluorescence could be detected on the sixth day after inoculation, which indicated that the conidia of *U. virens* had entered the glume from the outside. Some rice stigmas emitted bright green fluorescence under the blue light excitation of the fluorescence microscope, indicating that the rice stigmas were wrapped by the hyphae of *U. virens* (Figure 3).

#### 3.4.4. Wrapping of rice floral organs with *U. virens*

**FIGURE 4:**
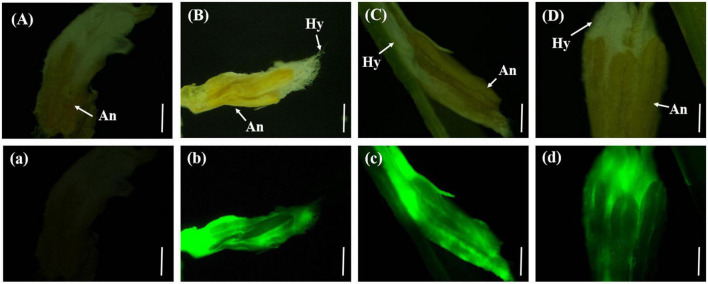
The hyphae of *Ustilaginoidea virens* wrap the whole floral organ. **(Aa)** UV-PD11 strains, **(Bb,Cc,Dd)** gfp-z1 strain. **(A–D)** Bright field, **(a–d)** dark field. Hy, hypha; An, anther. Bar, 500 μm.

On the eighth day after inoculation with gfp-z1 strain, the surface of stamen and pistil of rice flowers were covered by a large number of white hyphae, forming a flower hyphae complex. The strong fluorescence was observed under the fluorescence microscope, which indicated that the hyphae of *U. virens* had mainly gathered in the middle and lower parts of the stamens and had continuously extended to the top of the anthers. The development of rice floral organs was affected by *U. virens*, the anthers were shriveled, and the elongation of filaments was inhibited, which led to the failure of normal rice flowering (Figure 4).

#### 3.4.5. Fluorescence was detected on rice false smut balls

**FIGURE 5:**
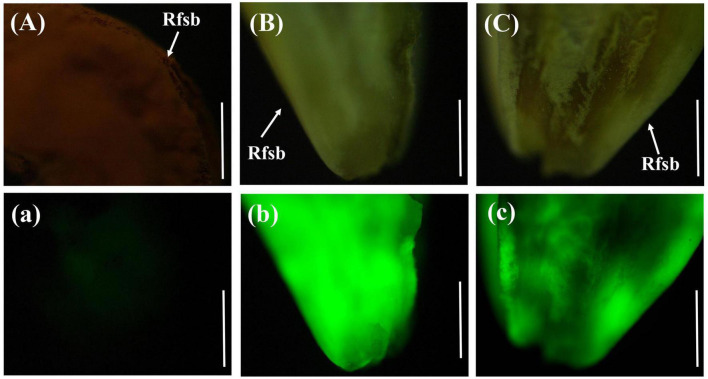
Green fluorescence emitted from the whole surface of rice false smut ball. **(Aa)** UV-PD11 strains, **(Bb,Cc)** gfp-z1 strain. **(A–C)** bright field, **(a–c)** dark field. Rfsb, rice false smut ball; Bars, 25 mm **(Aa)**, 200 μm **(Bb,Cc)**.

The rice anthers had seriously withered 12 days after inoculation with gfp-z1 strain, and a large number of hyphae of *U. virens* clustered in the lower part of the anthers, whereas only a few hyphae clustered in the upper part. After 15 days, rice false smut balls were clearly visible to the naked eye. Bright green fluorescence could be detected in the whole rice false smut balls excited by blue light, whereas the control group rice inoculated with unlabeled *U. virens* exhibited no fluorescence (Figure 5).

### 3.5. Anatomical observation of rice false smut balls

**FIGURE 6:**
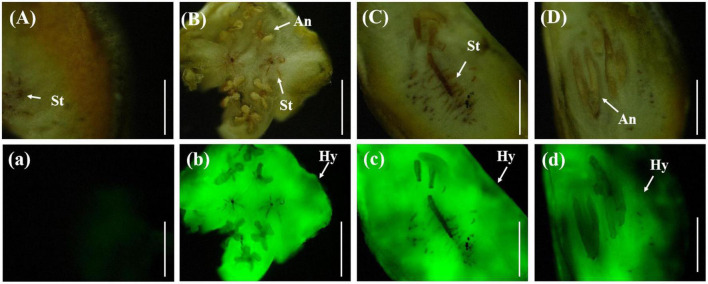
Anatomical images of transverse and longitudinal cuttings of rice false smut ball. **(Aa)** UV-PD11 strains, **(Bb,Cc,Dd)** gfp-z1 strain. **(A–D)** Bright field, **(a–d)** dark field. St, stigma; Hy, hypha; An, anther. Bar, 25 mm.

In the horizontal and vertical sections of the rice false smut balls, the anthers and stigma of rice were clearly visible. The anthers were butterfly-shaped and the stigma was radial, all of which were tightly embedded by the white hyphae of *U. virens*. Under the fluorescence microscope, strong fluorescence could be detected in the infected area. The stigma and anther were tightly wound by *U. virens*, and the stigma was brown when the rice false smut ball was transected (Figure 6).

### 3.6. Formation process of rice false smut balls

**FIGURE 7:**
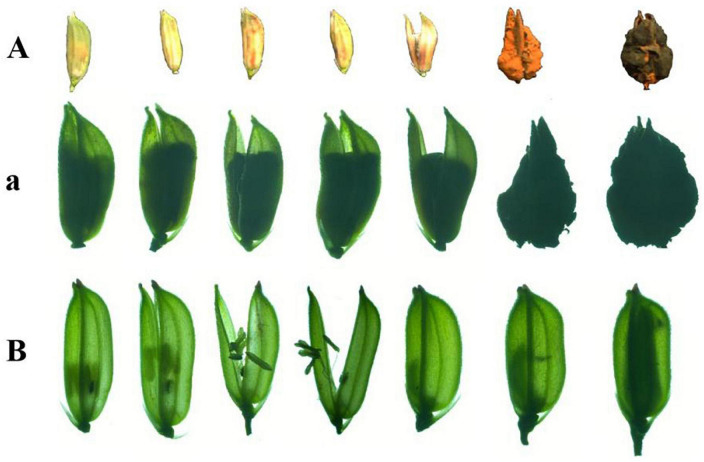
The formation process of rice false smut balls. **(A,a)** The main process of rice false ball formation and **(B)** normal pollination process of rice.

*U. virens* inoculated rice spikelets and uninoculated rice spikelets were sampled, observed under a stereoscope, and photographed to record the formation process of rice false smut balls. The entire floral organ of rice was wrapped by *U. virens*, which led to an inability of the rice to bloom normally. A massive structure (floral organ – *U. virens* complex) was observed under the stereoscope, which gradually expanded and finally opened the glumes, forming rice false smut balls that were two to three times larger than those observed in normal uninfected rice. The rice false smut ball was bright yellow at the early stage, which then turned into yellow-green and dark green at the later stage. Meanwhile, the chlamydospore on the surface of the rice false smut balls changed from a nondormant to a dormant state. The floral organs of normal rice grains were scattered, the anthers, filaments, and stigma were clearly visible under a stereomicroscope. At the later stage, the filaments stretched, the glume expanded and then extended outward, the anthers burst, and the pollen fell off and scattered randomly on floral organs, such as rice stigma and filaments. After pollination, the glume closed and the successfully pollinated ovary gradually expanded and filled (Figure 7).

### 3.7. Reisolation of the strain gfp-R1

**FIGURE 8:**
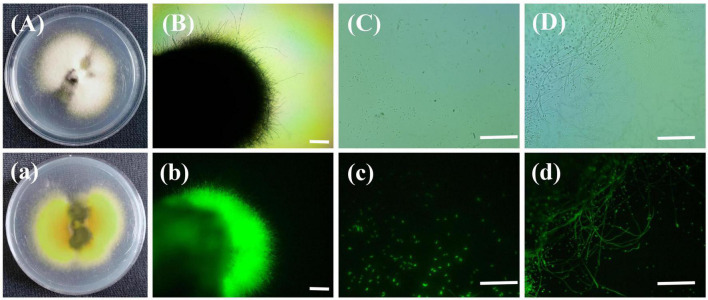
Colony of the strain gfp-R1 and morphology of hyphae and spores. **(Aa,Bb)** Colony of the strain gfp-R1, **(Cc,Dd)** morphology of hyphae and spores of *Ustilaginoidea virens*. **(A–D,a)** Bright field, **(b–d)** dark field. Bar, 50 mm.

The rice false smut balls with green fluorescence were isolated to obtain a strain of *U. virens*. As shown in Figure 8, the colonies of the obtained *U. virens* were white at the early stage and pale yellow at the late stage. The back of the colony was orange and the center of the colony was dark green, which were the typical characteristics of *U. virens* at the late stage. Observation of the strain under a fluorescence microscope revealed that the colony, conidia, and hyphae of the strain gfp-R1 emitted bright fluorescence. The obtained gene encoding green fluorescent protein was stably inherited and expressed in *U. virens*. This indicated that rice false smut produced via inoculation with the strain labeled by green fluorescent protein could be preserved, so that the *U. virens* with strong pathogenicity and expressing gfp could still be obtained at the later stage.

### 3.8. The infection process of *U. virens*

**FIGURE 9:**
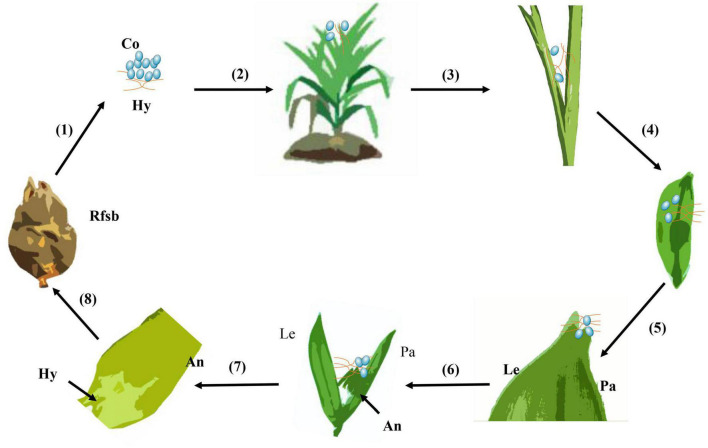
A schematic of the infection process of *Ustilaginoidea virens*. (1) Chlamydospore germination, (2) chlamydospores adhered to the surface of rice, (3) mycelia and conidia of *U. virens* entered the panicle bud, (4) the conidia of *U. virens* germinated on the glume surface after about 12 h, (5) numerous hyphae of *U. virens* clustered in the gap between the palea and lemma in rice after about 3 days, (6) rice floral organs were infected by *U. virens* after about sixth day, (7) the floral organ of rice formed a complex with the mycelium of *U. virens* after about eighth day, and (8) *U. virens* hijacked rice nutrition to form a rice false smut ball. Rfsb, rice false smut ball; Co, conidia; Hy, hypha; An, anther; Le, lemma; Pa, palea.

Rice false smut could be produced through inoculation of rice via panicle sheath instillation at stages 6–8 of rice young panicle differentiation (as shown in Figure 9), which could potentially explain the natural infection pathway of *U. virens* in the field. Based on findings in this study, a schematic of the process of *U. virens* infection was constructed (Figure 9). The conidia of *U. virens* were produced from sclerotia or chlamydospore, which became an important infection source. In the field, during the sixth to eighth stages of rice young panicle differentiation, the conidia of *U. virens* could enter the panicle bracts through the panicle sheath gap via rain or dew. The conidia of *U. virens* germinated on the surface of rice hulls to produce hyphae, which entered the glumes through the gap between the palea and lemma. *U. virens* randomly attached to the surface of the floral organs, causing pollen abortion, and absorbed nutrients from the rice floral organs to form rice false smut balls.

## 4. Discussion

In recent years, many reports on rice false smut caused by *U. virens* in rice-growing areas worldwide have been published (Wang et al., 2016). Nevertheless, when and how the conidia of *U. virens* enter the rice spikelets in the field remains unclear (Qiu et al., 2019). To characterize the natural infection process of *U. virens*, green fluorescent protein-labeled *U. virens* was used as the inoculum to inoculate rice via the nondestructive panicle sheath drip irrigation method, so that the entire infection process of *U. virens* could be clearly visualized. The results showed that *U. virens* germinated on the glume surface of rice and entered the rice via the gap between the palea and lemma. The pathogen colonized the floral organ, hijacked the nutrients of the rice, and formed rice false smut balls, which were two to three times larger than those observed in normal uninfected rice. The results of this study provide a reference for the formulation of strategies to control rice false smut in the field.

The panicle sheath of rice is relatively sealed and plays an important role in preserving heat and retaining moisture, while facilitating the germination and colonization of *U. virens* on the surface of the rice glume (Hu et al., 2014, 2021). Therefore, the natural process of *U. virens* infection might occur before the breakage of rice young panicle. After 3 days of inoculation with *U. virens*, bright fluorescence appeared between the palea and lemma of rice, indicating that a large number of hyphae of *U. virens* had clustered there, which promoted the attachment and colonization of *U. virens* due to the special structure of the palea and lemma. Observation of the sections of rice glume showed no fluorescence in the outer skin cells, which indicated that it was difficult for *U. virens* to directly penetrate the glume epidermis into the glume. Meanwhile, studies have shown that *U. virens* colonizes rice roots and mainly grows in intercellular spaces but has difficulty directly penetrating cell walls, which is consistent with the results obtained in the present study (Prakobsub and Ashizawa, 2017; Yong et al., 2018). After 6 days of inoculation with *U. virens*, fluorescence was detected in a few stigmas, indicating that *U. virens* had successfully entered the rice glume. In addition, the hyphae of *U. virens* mainly clustered in the middle and lower stamens and continuously extended to the top of the anthers, which affected anther development. Inhibition of the elongation of filaments led to the failure of normal flowering of rice. Researchers found that *U. virens* mainly colonized the base of rice filaments, because the gap between filament cells was large, which aided the propagation and infection of pathogens (Fan et al., 2015; Song et al., 2016). It is worth noting that the hyphae of *U. virens* extend along the intercellular space of the filaments instead of penetrating the cell wall of rice (Tang et al., 2013). To identify the source of nutrients used for the formation of rice false smut balls, researchers found that the balls could be divided into three layers from interior to exterior. The innermost layer is composed of white hyphae, the middle orange-colored layer is composed of hyphae and chlamydospores, and the outermost layer is composed of chlamydospores (Meng et al., 2015). The pistils and stamens of rice were embedded by the hyphae of *U. virens*. The ovary was full and emerald green at the early stage and dry and dark brown at the late stage. Therefore, it was speculated that the rice ovary may be the main source of nutrients for the formation of rice false smut balls (Hu et al., 2021). In this study, bright fluorescence was observed around the floral organ, which indicated that the ovary, filament, and anther of rice were wrapped by the hyphae of *U. virens*. There was no fluorescence in the anther, pollen, and stigma, which provides new evidence that *U. virens* cannot penetrate the floral organs.

In this study, the main infection process of *U. virens* was revealed through careful observation and recording, thereby providing a theoretical basis for the formulation of strategies to control rice false smut in the field.

## Data availability statement

The original contributions presented in this study are included in the article/Supplementary material, further inquiries can be directed to the corresponding authors.

## Author contributions

ML, RL, and XH: conceptualization. XH, XW, and JW: investigation. XH, YZ, and JW: methodology. YZ: software. XH and JW: writing—original draft preparation and writing—review and editing. All authors had read and agreed to the published version of the manuscript.
