# Supplementary material for: Visualization of the entire process of rice spikelet infection by Ustilaginoidea virens through nondestructive inoculation
Source: Front Microbiol. 2023 Aug 10;14:1228597. doi: 10.3389/fmicb.2023.1228597 (PMC10450503; doi:10.3389/fmicb.2023.1228597)
Supplement: Supplementary file 1 [file Data_Sheet_1.PDF]

Supplementary Material

Visualization of the Entire Process of Rice Spikelet Infection by *Ustilaginoidea virens* Through Nondestructive Inoculation

Xianfeng Hu, Jian Wang, Yubo Zhang, Xiaomao Wu, Rongyu Li, and Ming Li

*** Correspondence:** lirongyu0328@126.com (Rongyu Li), lm21959@163.com (Ming Li).

1 Supplementary Figures

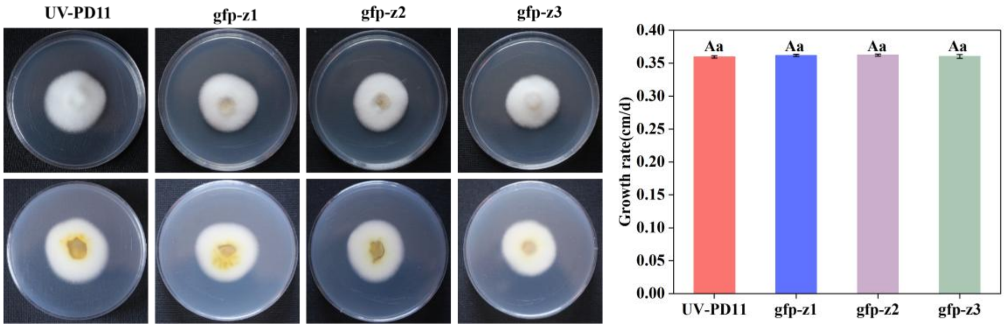

Supplementary Figure S1. Colony morphology and growth rate of strains UV-PD11, gfp-z1, gfp-z2, and gfp-z3.

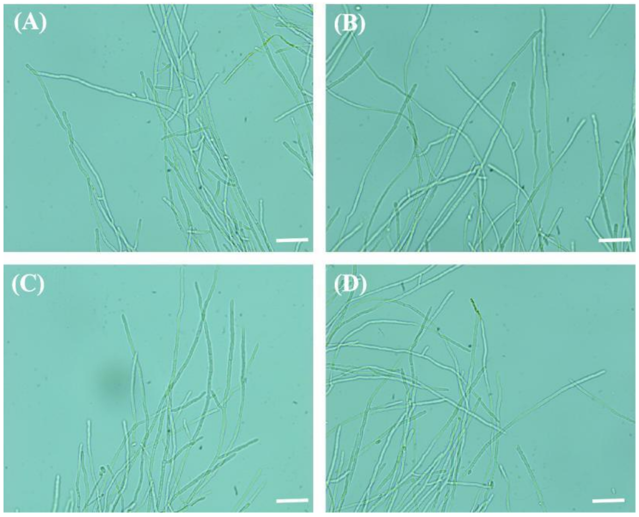

Supplementary Figure S2. Comparison of the hyphal morphology of the UV-PD11 strain and transformants. (A) UV-PD11 and (B-D) gfp-z1, gfp-z2, and gfp-z3, respectively. Bar, 50 μm.

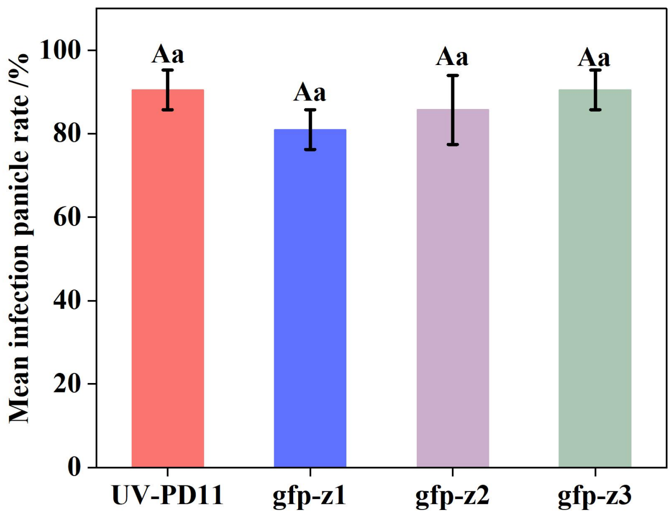

Supplementary Figure S3. Pathogenicity differences between the transformant and UV-PD11 strains.
